# Multicenter Comparative Study of Six *Cryptosporidium parvum* DNA Extraction Protocols Including Mechanical Pretreatment from Stool Samples

**DOI:** 10.3390/microorganisms8091450

**Published:** 2020-09-22

**Authors:** Nicolas Valeix, Damien Costa, Louise Basmaciyan, Stéphane Valot, Anne Vincent, Romy Razakandrainibe, Florence Robert-Gangneux, Céline Nourrisson, Bruno Pereira, Emilie Fréalle, Philippe Poirier, Loic Favennec, Frederic Dalle

**Affiliations:** 1 Laboratoire de Parasitologie-Mycologie, Plateforme de Biologie Hospitalo-Universitaire, 2 rue A. Ducoudray, BP 37013, CEDEX, 21070 Dijon, France; nicolas.valeix@chu-dijon.fr (N.V.); louise.basmaciyan@chu-dijon.fr (L.B.); stephane.valot@chu-dijon.fr (S.V.); anne.lamy@chu-dijon.fr (A.V.); 2 Laboratoire de Parasitologie-Mycologie, Centre Hospitalo-Universitaire C. Nicolle de Rouen, 76000 Rouen, France; damien.costa@chu-rouen.fr (D.C.); romy.razakandrainibe@univ-rouen.fr (R.R.); loic.favennec@chu-rouen.fr (L.F.); 3 Centre National de Référence–Laboratoire Expert des Cryptosporidioses, Institut de Biologie Clinique, Centre Hospitalo-Universitaire C. Nicolle de Rouen, 76000 Rouen, France; 4 UMR PAM, University Bourgogne Franche-Comté-AgroSup Dijon-Equipe Vin, Aliment, Microbiologie, Stress, CEDEX, 21078 Dijon, France; 5 Univ. Rennes, CHU Rennes, Inserm, EHESP, Irset (Institut de Recherche en Santé Environnement Travail), UMR_S 1085, 35000 Rennes, France; florence.robert-gangneux@univ-rennes1.fr; 6 Laboratoire de Parasitologie-Mycologie, Centre Hospitalo-Universitaire de Clermont-Ferrand, 63000 Clermont-Ferrand, France; c_nourrisson@chu-clermontferrand.fr (C.N.); bpereira@chu-clermontferrand.fr (B.P.); ppoirier@chu-clermontferrand.fr (P.P.); 7 CHU Lille, Laboratoire de Parasitologie-Mycologie, F-59000 Lille, France; emilie.frealle@chru-lille.fr; 8 Univ. Lille, CNRS, Inserm, CHU Lille, Institut Pasteur de Lille, U1019–UMR8204-CIIL-Center for Infection and Immunity of Lille, F-59000 Lille, France

**Keywords:** *Cryptosporidium parvum*, grinding, DNA extraction, stool samples, real-time PCR, molecular diagnosis

## Abstract

Background: Nowadays, many commercial kits allow the detection of *Cryptosporidium* sp. in stool samples after deoxyribonucleic acid (DNA) extraction. Protocols of stool pretreatment have been proposed to optimize oocysts’ DNA extraction. Among them, mechanical grinding was reported to improve the performance of Cryptosporidium oocysts’ DNA extraction. Methods: A multicenter comparative study was conducted within the framework of the French National Reference Center-Expert Laboratory for Cryptosporidiosis. Six extraction systems (i.e., manual or automated) associated with various mechanical pretreatment protocols, were compared for the *Cryptosporidium parvum* oocyst’ DNA extraction, before amplification using the same real-time PCR method targeting. Results: The sensitivity of real-time PCR assay was unequally impacted by the pretreatment/extraction protocol. We observed significant differences for the lowest concentrations of *C. parvum* oocysts (i.e., 0–94.4% and 33.3–100% respectively for 10 and 50 oocysts/mL). All in all, the protocol using Quick DNA Fecal/Soil Microbe-Miniprep^®^ manual kit showed the best performances. In addition, optimal performances of mechanical pretreatment were obtained by combining a grinding duration of 60 s with a speed of 4 m/s using Fastprep24^®^ with Lysing Matrix E^®^. Conclusions: Sample pretreatment, as well as the extraction method, needs to be properly adapted to improve the diagnostic performances of the *C. parvum* DNA amplification methods.

## 1. Introduction

The protozoan parasite *Cryptosporidium* sp. infects a wide range of vertebrate hosts, including humans, in whom it behaves as an opportunistic pathogen. In healthy people, cryptosporidiosis can be responsible for gastrointestinal symptoms, acute diarrhea being the most common clinical manifestation. However, severe manifestations can be observed, especially in immunocompromised patients (e.g., organ transplantation, HIV infection), including persistent diarrhea and extra-intestinal infections [1]. Among *Cryptosporidium* spp., *C. parvum* and *C. hominis* are the most widespread species isolated in humans, with varying prevalence worldwide [2,3]. Thus, *C. hominis* is the species most often isolated (i.e., 62% of cases on average) in North and South America, Africa, and Australia, while *C. parvum* is the species most often isolated (57% of cases on average) in Europe, particularly in the United Kingdom [2]. In France, these two species represent more than 90% of cases of cryptosporidiosis diagnosed [3]. Commonly, transmission to humans occurs by ingestion of oocysts excreted in the environment through contaminated food or water, or through a direct fecal-oral route. Thus, cryptosporidiosis is a current public health concern, with waterborne and foodborne outbreaks occurring each year worldwide [4]. In this context, efficient biological methods for cryptosporidiosis diagnosis are needed.

Nowadays, molecular diagnosis methods by polymerase chain reaction (PCR) are increasingly replacing the use of time-consuming microscopic techniques for the detection of *Cryptosporidium* sp. in stool samples [5]. A wide range of PCR techniques are currently available that are more sensitive and specific than conventional microscopic techniques. These include specific simplex PCR to the latest real-time multiplex PCR test detecting *Cryptosporidium* sp. with other intestinal parasites of interest [6,7,8]. Real-time PCR techniques allow performant detection and quantification of *Cryptosporidium* sp. DNA from stool samples [9]. However, this is a multi-step procedure, each step being influenced by various parameters. The oocyst wall is a robust, thick-wall structure composed of three layers of filamentous glycoproteins (Oocyst Wall proteins (OWPs)) and acid-fast lipids [10,11,12], which makes it difficult to extract DNA by conventional methods. Thus, the performances of PCR methods in detecting *Cryptosporidium* sp. DNA depends on the quality of the extracted DNA. Among the pretreatment methods developed to improve DNA extraction, mechanical grinding using ceramic or glass beads has been reported as a step improving the performance of the extraction [13,14]. This additional preliminary step is also critical and needs optimization for the efficient extraction of DNA from *Cryptosporidium* sp. oocysts.

Few studies have compared the performance of DNA extraction methods from *Cryptosporidium* sp. oocysts in stools or even in food specimens, most of them evaluating one or two methods [15,16,17,18]. Previous studies have already reported Nuclisens^®^ easyMAG^®^ (BioMérieux™) method with preliminary mechanical grinding as providing the best performances, confirming the importance of the grinding pretreatment in optimizing the performances of DNA extraction from *Cryptosporidium* sp. oocysts [14]. However, the influence of the multiple pretreatment parameters upon the DNA extraction performances was not investigated in this study.

Thus, knowing that (i) mechanical pretreatment has been proved to improve DNA extraction from *Cryptosporidium* oocysts’ [14] and that (ii) most of the manufacturers do not provide any recommendation for the mechanical pretreatment aimed at improving DNA extraction from parasites such as *Cryptosporidium* sp., most of the laboratories adapt and set up their own pretreatment phase.

In this context, a multicenter comparative study was conducted within the framework of the National Reference Center-Expert Laboratory for Cryptosporidiosis (CNR-LE Cryptosporidioses, University Hospital of Rouen, France), involving five university hospital laboratories of medical parasitology. The aims of this study were to compare the performances of different “pretreatment protocols in combination with manual or automated DNA extraction systems” routinely used by most of the clinical microbiology laboratories and displaying various protocols of mechanical pretreatment.

## 2. Materials and Methods

The study was conducted between 25 February to 17 March 2019 in five medical parasitology laboratories from the University Hospitals of Rouen, Rennes, Lille, Clermont-Ferrand, and Dijon, with recognized proficiency in the molecular detection of *Cryptosporidum* sp.

### 2.1. Design of the Study

In order to study the impact of extraction protocols for *C. parvum* DNA amplification’, fourteen stools samples, with various concentration of oocysts, ranging from 0 to 1000 oocysts/mL, were tested per protocol tested. The performances of the extraction protocols were first evaluated by comparing the average percentage of positive *C. parvum* PCRs over the total number of PCRs performed at each *C. parvum* oocysts’ concentration for a given method as described elsewhere [19].

### 2.2. Mimic Stool Samples Preparation

The CNR-LE Cryptosporidioses (University Hospital of Rouen, France) provided oocysts of *Cryptosporidium parvum* (*C. parvum*) from diarrheal feces initially isolated from a child. The coordinating center (University hospital of Dijon, France) prepared stool samples seeded with oocysts of *C. parvum*. A human feces negative for common digestive parasites by microscopy and for *Cryptosporidium* sp. and *Giardia duodenalis* by PCR methods was used as the seed matrix. Type 7 stools according to the Bristol Stool Form Scale (BSFS) were prepared from this stool according to the following protocol: 20 g of stools in 50 mL of physiological saline (0.09% NaCl), filtered through a large mesh strainer and stored at 4 °C. The concentration of the stool suspension supplied by the CNR-LE for Cryptosporidioses was approximately 1 × 10^6^ oocysts/mL. Dilutions were made in the dilution stool to obtain 6 stool suspensions at final concentrations of, 1000, 500, 100, 50, 10, and 0 oocysts/mL.

The number of DNA extractions varied with the parasite concentration tested and was higher for the lowest concentrations (a maximum of 3 extractions was carried out at the 10 oocysts/mL concentration) (Table 1). For each of the extraction protocols tested, 14 stool samples containing 0 (*n* = 1), 10 (*n* = 3), 50 (*n* = 3), 100 (*n* = 3), 500 (*n* = 2), 1000 (*n* = 2) oocysts/mL were prepared.

The stool samples were sent at 4 °C within 24 h to the participating laboratories and were stored at 4 °C until the experiment.

### 2.3. Mechanical Pretreatment

Mechanical grinding was carried out using beads of different chemical compositions (glass, ceramic, or garnet beads) with diameters ranging from 0.074 to 1.6 mm depending on the lysis matrices provided by the manufacturers. Four brands of homogenizers with oscillating movement and a vortex homogenizer were used for the pretreatment step (Table 2).

### 2.4. Impact of Grinding Parameters on DNA Extraction Efficiency

The impact of the pretreatment step on the performances of the extraction of *Cryptosporidium* DNA from stool specimens were investigated for the lowest oocyst concentrations (i.e., 5, 10, and 50 oocysts/mL of stool) adapting the protocols from You et al., 2014 and Cha et al., 2014 [20,21]. Briefly, the conditions tested were 4, 5, and 6 m/s for the speed grinding and 30, 60, and 120 s for the grinding duration. We carried out these tests on the FastPrep 24^®^ grinder/homogenizer (MP Biomedicals™) with Lysing Matrix E^®^ (MP Biomedicals™), then extracted the stool shreds on Nuclisens^®^ easyMAG^®^ (bioMérieux™). Finally, *Cryptosporidium* DNA detection in stool extracts was carried out by PCR using our in-house method (see below). One extraction was performed for each combination of speed and duration of grinding parameters and 6 PCRs were carried out per DNA extract, corresponding to a total of 162 PCRs.

### 2.5. DNA Extraction

All participating laboratories received the 14 stool samples and an internal control on the same day (Table 1). DNA extractions from stool samples were performed in each center within seven days after receipt according to the manufacturer’s instructions of each extraction system tested. Six pretreatment/extraction protocols were evaluated, including three manual extraction systems and three automated extraction systems (Table 3). All extraction protocols were systematically associated with a mechanical pretreatment with beads, and one method (MP) was also evaluated without mechanical grinding.

Three automated DNA extraction systems were tested in three laboratories: (a) the ELITE InGenius^®^ system (IN) (ELITechGroup Molecular Diagnostics, Puteaux, France), using the extraction cartridges ELITe InGenius^®^ SP200 (b) MagnaPure 96 System^®^ (Roche Diagnostics, Boulogne-Billancourt, France) using the MagNA Pure 96 DNA and Viral NA Small Volume^®^ extraction kit, and (c) the NucliSens easyMAG^®^ system (EM) using the NucliSens silice magnet (bioMérieux, Craponne, France) extraction kit. MagnaPure 96 System^®^ was the only extraction system tested without (MP), and with grinding (MPG). Three manual extraction systems were tested: QIAamp Fast DNA Stool Mini Kit^®^ (QF) and QIAamp Power Fecal DNA Kit^®^ (QP) (Qiagen, Venlo, The Netherlands) Quick DNA Fecal/Soil Microbe Miniprep^®^ (ZR) (ZymoResearch, Irvine, CA, USA). After pretreatment, ten μL of internal control (CEI) was added to each sample before extraction (Diagenode DiaControlDNA ™ DNA virus marked with Yellow Dye (ref DICD-YD-L100)). The CEI is a complete DNA of phocid herpes. It has no sequence similarity to the human genome, to other viruses or other infectious pathogens.

Extraction was performed according to the manufacturer’s recommendations (Table 3). DNA extracts were then stored and sent at 4 °C to the coordinating center (University hospital of Dijon, France) for PCR amplification of *Cryptosporidium* DNAs. The PCRs were performed within 15 days after receipt.

### 2.6. DNA Amplification

A total of 960 PCRs was carried out, including 700 *Cryptosporidium*-specific PCRs for *Cryptosporidium* DNA detection in stool extracts, 98 PCRs for the internal control detection in stool extracts and 162 *Cryptosporidium*-specific PCRs for the additional study aims at evaluating the impact of grinding parameters on DNA extraction efficiency.

### 2.7. Cryptosporidum-Specific PCR

An in-house PCR was used for the detection of *Cryptosporidium* DNA in stool samples as previously described [22]. Briefly, *Cryptosporidium* DNA amplification targeted a 258-pb fragment located in the 18S ribosomal ribonucleic acid (rRNA) gene and was performed by real-time PCR, using FRET probes detection on a LightCycler 2.0 system (Roche Diagnostics). PCR amplification and detection of *Cryptosporidium* DNA was carried out using the forward 5′-GTT AAA CTG CRA ATG GCT-3′ (Cry80F3) and reverse 5′-CGT CAT TGC CAC GGT A-3′ (Cry337R) primers with the hybridization probes: 5′-CCG TCT AAA GCT GAT AGG TCA GAA ACT TGA ATG-3′ Fluorescein (anchor probe) and 5′-Red 640-GTC ACA TTA ATT GTG ATC CGT AAA G 34 Phosphate (sensor probe). Primers and probes were used at a concentration of 10 μM. Five microliters of DNA extracts were added to a final reaction volume of 20 μL to each amplification reaction tube.

### 2.8. Inhibitor Detection and Control Extraction

We used the commercial kit DiaControlDNA^TM^ (Diagenode) as external control of inhibition (CEI) aimed at validating the extraction and detecting PCR inhibitors. CEI was detected according to the manufacturer’s recommendations in all the samples tested, indicating that all six protocols tested performed well in extracting DNAs and in eliminating PCR inhibitors.

### 2.9. Statistical Analysis

The optimal number of DNA extractions and DNA amplifications for each concentration were determined according to Poisson’s law as described elsewhere [19,23]. The frequency of positive PCRs obtained with the various protocols were compared using the chi-square test. In cases of small sample sizes, Fisher’s exact test was used. The Ct values found by PCR were compared among the different protocols using Kruskal–Wallis test, followed when appropriate (omnibus *p*-value less than 0.05) by Dunn’s two by two post-hoc test. A probability of 0.05 or less was considered to be significant.

## 3. Results

### 3.1. Influence of Pretreatment/Extraction Protocol on C. parvum DNA Amplification

All the negative controls included in the study were negative by PCR. For the spiked samples, the performances in *C. parvum* DNA amplification were variable depending on the protocol used. The protocol with the manual extraction system ZR showed the best performances with an average positivity rate of 98.9% (Table 4). At the opposite, the protocol using the QF manual extraction system had the lowest performances, with an average positivity rate of 56.7%. The other protocols included in this study displayed acceptable performances with average positivity rates ranging from 80% to 94.4% (Table 4).

When analyzing the results depending on the *C. parvum* concentration, a significant difference was found among the protocols when extracting samples containing ≤100 oocysts/mL. Protocols with QF displayed the worst performances compared to all other protocols (*p* ≤ 0.001) at the concentrations of 10, 50, and 100 oocysts/mL (*p* ≤ 0.05 to *p* ≤ 0.001). At the concentration of 50 oocysts/mL, the protocols yielded 33.3 to 100% of positive PCR results (Table 4): protocols using EM and ZR displayed significantly better performances compared to protocols QF, MP, and IN protocols (Table 5). At the lowest concentration of 10 oocysts/mL, the protocols yielded 0 to 94.4% of positive PCR results: only QF protocol showed significantly lower performance than all other protocols (0% versus 44.4–94.4%) (*p* ≤ 0.01 to *p* ≤ 0.001).

For samples containing >100 oocysts/mL, as all the protocols tested reached average positivity rates of 100%, a comparison of the performances of the protocols was investigated considering the average cycle threshold (Ct) values obtained during *C. parvum* DNA amplification by PCR at a given oocysts’ concentration for each of the protocols tested (Table 6). At the concentration of 1000 oocysts/mL, the mean Ct values ranged from 28.89 ± 0.37 to 35.27 ± 0.84 with significant differences (*p* ≤ 0.001) among protocols except between EM and MPG. At the concentration of 500 oocysts/mL, the mean Ct values ranged from 29.86 ± 0.16 to 33.23 ± 0.44 with significant differences (*p* ≤ 0.001) among all protocols except between EM and QP and between IN and MP. Interestingly, when comparing the MP protocol with or without grinding as a pretreatment, significant performance differences were observed between MP and MPG at the highest concentrations of 500 and 1000 oocysts/mL (*p* ≤ 0.001) (Table 7).

All in all, the ZR extraction protocol showed the best performances in this study, followed by EM, MPG, and QP protocols. Finally, the IN and QF protocols had the lowest extraction performances.

### 3.2. Performances in C. parvum DNA Amplification Depend on the Pretreatment Protocol

**Figure 1:**
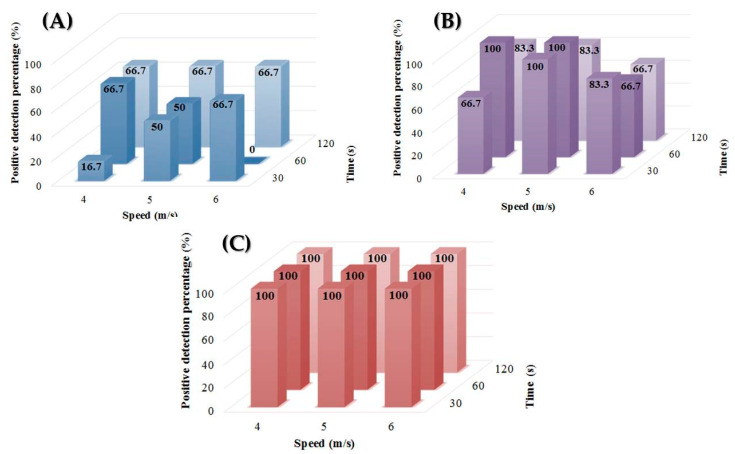
The proportion of positive samples (%) according to the speed and duration of mechanical pretreatment for the concentration of (**A**) 5 oocysts/mL, (**B**) 10 oocysts/mL, and (**C**) 50 oocysts/mL.

The largest differences between the extraction protocols having been obtained for the lowest oocyst concentrations (i.e., 10 and 50 oocysts/mL), we therefore investigated the impact of the speed and duration of the grinding step on extraction performances at low oocyst concentrations (i.e., 5, 10 and 50 oocysts/mL). Several combinations of grinding speed and duration with the Lysing Matrix E^®^ (MP Biomedicals™) were tested on a FastPrep 24^®^ grinder/homogenizer (MP Biomedicals™). Optimizing this step is essential in order to avoid potential DNA fragmentation and the release of PCR inhibitors. Differences in DNA extraction performances were observed depending on the mechanical pretreatment protocol used, especially at the concentrations of 5 and 10 oocysts/mL (Figure 1). The optimal performances were indeed obtained when combining a grinding duration of 60 s with a speed of 4 m/s. Finally, no difference was observed at concentrations > 50 oocysts/mL. Above this concentration, the variation of these parameters seems to no longer have any influence on the extraction performances. Thus, from a given concentration of oocysts/mL, the amount of amplifiable DNA, even qualitatively altered by a potential fragmentation, would no longer influence the positive percentage of PCR detection.

## 4. Discussion

Molecular techniques for the detection of *Cryptosporidium* sp. DNA in stool specimens are currently available that allow rapid and specific diagnosis of cryptosporidiosis in humans. Since few oocysts are sufficient to cause infections associated with clinical symptoms [24,25,26], sensitive molecular tests are necessary to detect low oocyst concentrations in stool specimens. Nowadays, diagnostic methods present variable limits of detection (i.e., from 50 to 500 oocysts/mL for DNA amplification methods [14,16,27,28], 1000 oocyst/mL for auramine phenol stain, 6000 oocysts/mL for immunofluorescence and 3 × 10^5^–10^6^ oocysts/mL for modified Ziehl-Neelsen stain and antigen detection enzyme-linked immunosorbent assay (ELISA) [29,30,31]).

Various studies have already shown that the real-time PCR assays targeting 18S ribosomal DNA *Cryptosporidium* sp., display variable performances in terms of sensitivity and/or specificity [14,16]. However, evaluation of the DNA extraction step, which is critical for providing a sufficient amount of DNA with high-grade quality and purity for optimal PCR amplification, has been poorly investigated. A previous study evaluated six commercial systems for *Cryptosporidium* sp. DNA extraction (including two automated systems and four manual systems), reporting the best performances in extracting *Cryptosporidium* sp. DNA for the Nuclisens^®^ easyMAG^®^ (bioMérieux™) system is associated with mechanical grinding as a pretreatment step [14]. This highlighted the importance of the mechanical pretreatment in the overall performances of the extraction system. However, the influence of the technical parameters of this mechanical pretreatment on the performances of *Cryptosporidium* sp. DNA extraction has been poorly investigated. Thus, the aims of our study were to compare the performances of (i) recent manual and automated DNA extraction systems routinely used in clinical microbiology laboratories, and of (ii) various mechanical pretreatment protocols to improve the efficiency of extraction of *C. parvum* oocysts in stool specimens. The main originality of our study resides in the methodology used to ensure an unbiased evaluation of the performance of DNA extraction. First, all DNA extractions were performed using stool suspensions of fresh oocysts, within a short period of time (<7 days) [32] to avoid the deleterious effect of long-term storage on the viability and integrity of *Cryptosporidium* sp. oocysts [33,34,35]. In addition, all PCRs were performed within 15 days on DNA extracts kept at 4 °C, avoiding freezing/thawing of DNA that can impair DNA integrity and its further PCR amplification, as demonstrated by Fayer et al., 1996 and Yera et al., 2009 [19,36,37]. Finally, the numbers of DNA extractions and DNA amplification for each concentration and protocols tested were optimized according to the Poisson’s law as described elsewhere [19,23], to warrant the robustness of the statistical analyses in this comparative study.

Six recent DNA extraction systems, with three automated systems (i.e., IN, EM, and MPG) and three manual systems (i.e., QF, QP, and ZR) including a mechanical pretreatment step, were evaluated for their suitability to isolate *Cryptosporidium* sp. DNA from stool specimens. Whether considering the percentage of positive detection for concentrations ≤ 100 oocysts/mL or the Ct values for concentrations >100 oocysts/mL of stool, the best performances were obtained with Quick DNA Fecal/Soil Microbe Miniprep^®^. These data corroborate other observations that reported ZR Fecal DNA Miniprep kit^®^, a previous version of the kit evaluated herein, as exhibiting excellent performances in *Cryptosporidium* sp. DNA extraction with high extraction yields and a low proportion of PCR inhibitors are attributed to optimal purity and integrity of the DNA extracted by this kit [16]. In comparison, QIAamp Fast DNA Stool Mini Kit^®^ displayed the poorest performances, and no DNA amplification was observed for oocyst concentrations < 50 oocysts/mL of stool. The other protocols with mechanical pretreatment reported substantially good performances for *Cryptosporidium* sp. DNA extraction and amplification from stool specimens. However, slight differences in their respective performances were noticeable without the possibility to establish a clear classification of these protocols with regard to their performances. Indeed, despite the lack of statistical significance between the average percentages of positive detection for the EM, MPG, and IN protocols at the concentrations of 100, 500, and 1000 oocysts/mL of stool, significant differences were observed for these protocols considering the average Ct values for PCR detection at the same concentrations (Table 6 and Table 7). Interestingly, it is important to note that all the protocols tested would allow acceptable *Cryptosporidium* sp. DNA detection if the DNA extracts were routinely tested at least in triplicate since ≥33.3% of the PCR were positive at the concentration of 50 oocysts/mL for all the protocols (Table 4). Similar data were observed at the concentration of 10 oocysts/mL except for the QF protocol that did not allow DNA detection at this concentration.

When focusing on the protocols using an automated extraction system, EM, MPG, and IN provided good performances in extracting *Cryptosporidium* sp. DNA, even at low oocyst concentrations, since >72% and >44% of positive PCR results were obtained at the concentration of 50 and 10 oocysts/mL of stool, respectively, for all the three protocols with automated extraction system tested (Table 4). Interestingly, the IN protocol, that is based on Magtration technology, performed less efficiently in extracting *Cryptosporidium* sp. DNA compared to EM and MPG (Table 4). This corroborates other observations reporting high performances of Nuclisens easyMAG (Biomerieux) and MagNA Pure Compact (Roche Diagnostics) systems and low performances of the Magtration system 12GC (Precision System Science) in extracting *Toxoplasma gondii* DNA from amniotic fluids [23].

Considering that the complex physicochemical features of the oocyst wall make *Cryptosporidium* sp. DNA extraction difficult by conventional methods, several protocols of pretreatment have been developed, aimed at improving *Cryptosporidium* sp. DNA extraction. Among them, mechanical grinding by the use of ceramic or glass beads was reported to improve the performances [13,14,15,16,17,18]. Interestingly in our study, some observations strongly support the view that mechanical pretreatment of stool samples improved the quantity and/or the quality of extracted DNA from *Cryptosporidium* sp. oocysts. Here, the protocol associating QF system with mechanical pretreatment displayed a detection threshold of >50 oocysts/mL (Table 4), while it was 100-fold higher in the study by Mary et al. >5000 oocysts/mL), who did not apply any mechanical pretreatment [14]. Additionally, as a participating center routinely used MP extraction method without mechanical pretreatment, we consequently decided to include a comparison of MP versus MPG as a proof of concept of the need for mechanical pretreatment for improving DNA extraction from *Cryptosporidium* sp. oocysts. As expected, our data suggest that the protocol using MagnaPure 96^®^ with grinding performed better than MP, since significant differences in the mean Ct values were observed between the two protocols for concentrations > 100 oocysts/mL, with a difference of 2.36 Ct in favor of MPG (Table 7) (for concentrations ≤ 100 oocysts/mL, even if not statistically significant, the average percentage of detection was higher for MPG (100%) compared to MP (88.9%) (Table 4)). Collectively, these data suggested that the grinding protocol per se could influence the performances of the overall extraction protocol. To illustrate these observations, we further investigated the influence of the speed and duration of the grinding step on the extraction performances of one of the methods investigated in the present work, namely the EM system that combines the grinder Fastprep 24 (MP Biomedicals™) with Lysing Matrix E (MP Biomedicals™). Optimal performances were obtained when combining a duration of 60 sec with a speed of 4 m/s. Overall, in view of our data, it is difficult to conclude whether the discrepancies observed in the performances of the different combinations of mechanical pretreatment/extraction kits tested in our study were due to non-optimized mechanical pretreatment or to the lack of sensitivity of the commercial extraction kits per se. However, our data strongly suggested that (i) mechanical pretreatment is necessary for improving DNA extraction from *Cryptosporidium* sp. oocysts and (ii) optimizing the parameters of the grinding step could improve the performances of commercial kits in extracting DNA from *Cryptosporidium* sp. oocysts.

Optimized extraction protocols combined with sensitive and specific PCR assays are necessary to guarantee the detection of low loads of *Cryptosporidium* sp. oocysts in stool specimens. It is thus crucial to adapt the mechanical pretreatment step. First, the type of grinder could play a role in the process. Indeed, considering the oocyst wall of *Cryptosporidium* sp. as a composite material, the direction of applied forces is essential. Thus, the type of grinder will influence the disruption of the oocyst wall (horizontal oscillation, vertical oscillation, or vortexing). Additionally, the physicochemical characteristics of the beads can also influence the success of the pretreatment step. First, the size of the beads is essential. Indeed, the use of smaller beads would be more effective for the lysis of small structures, such as viruses, spores, or even oocysts. Some studies [20,21] which have tackled this problem in the context of the extraction and excystation of *Eimeria* sp. oocysts have shown that the use of small glass beads (0.5 mm) effectively broke the wall of oocysts while those of larger size preserved the sporozoites. Second, the shape of the grinding particles is a determining factor in the modality of cell rupture. Indeed, the balls of spherical shapes will lead to mechanical lysis by crushing forces, while the more irregular particles will complete the lysis by shearing forces [20,21]. Finally, the chemical composition of the beads determines two essential qualities for grinding: hardness and relative density; the first must be greater than the hardness of the wall of the oocyst and the second must be higher than the density of the buffer used, so that the beads do not float. In the light of these remarks, we suggest that the various performances observed in our study could partly be attributed to the different mechanical pretreatment protocols, since each of the protocol tested uses a particular combination of the type of grinder/speed and duration of grinding/size and composition of beads (Table 2). Larger studies are necessary to (i) specify the influence of each of these parameters, and (ii) standardize extraction protocols for *Cryptosporidium* sp. DNA from stool, especially in the context of low parasite loads.

In addition, the consistency of the stool, as well as its composition, may also bias the performance of extraction systems [14,38,39]. We indeed used in our study *Cryptosporidium* sp. oocyst suspensions of spiked liquid human stools (type 7 stools according to the BSFS), that was poor in fibers and residues since stool was formerly passed through a wide mesh colander. The reasons for using such liquid suspensions were (i) the ease of spiking at the desired concentrations, and (ii) the mimicking of the usual clinical samples (i.e., diarrheal stools as part of an explicit search for *Cryptosporidium* sp.).

## 5. Conclusions

Nowadays, many commercial kits allow the detection of *Cryptosporidium* sp. DNA in stool specimens. Given the complex physicochemical features of *Cryptosporidium* sp. oocyst wall, conventional DNA extraction systems provide poor performances in extracting *Cryptosporidium* DNA from the stool. Here, we showed that all the protocols tested in this study performed not equally efficiently in extracting *Cryptosporidium* sp. DNA from stool samples, especially at low oocyst concentrations (<100 oocysts/mL). We provided evidence that the three automated systems tested (i.e., EM, MPG, and IN) here were suitable for efficient extraction of *Cryptosporidium* sp. DNA even at low oocysts concentrations, provided that (i) a mechanical pretreatment is applied, and (ii) PCR is performed at least in duplicate on DNA extracts.

## Figures and Tables

**Table 1:** Design of the study: number of DNA extractions, *Cryptosporidium*-specific PCRs, internal control PCRs for the detection of inhibitors, according to the parasite concentration for each protocol.

Stool Concentration (oocysts/mL)	No. of Extractions Done Per Protocol	No. of *Cryptosporidium*-Specific PCRs		No. of Internal Control PCRs for the Detection of Inhibitors	
		Per Extraction	Total	Per Extraction	Total
0	1	2	2	1	1
10	3	6	18	1	3
50	3	6	18	1	3
100	3	6	18	1	3
500	2	11	22	1	2
1000	2	11	22	1	2
All	14		100		14

**Table 2:** Characteristics of mechanical pretreatment according to the DNA pretreatment/extraction protocol used by each participating center.

Participating Center	Center 1	Center 2		Center 3	Center 4	Center 5
Abbreviated Name of DNA Extraction System	EM	IN	QF	QP	ZR	MPG
Commercial name of mechanical lysis matrix (company)	Tube Lysing Matrix E^®^ (MP Biomedicals)	GB05^®^ (Next Advance) in 1.5 mL polypropylene tube (Eppendorf)		BeadTubes^®^, QIAamp PowerFecal DNA kit^®^ (Qiagen)	R BashingBead Lysis Tube^®^, kit Quick DNA Fecal/Soil Microbe Microprep kit^®^ (ZymoResearch)	MagnaLyser Green Tubes^®^ (Roche Diagnostics)
Diameter of beads	Mix of three types beads: −1.4 ± 0.2 mm ceramic spheres (64% ZrO_2_, 33% SiO_2_) −0.112 ± 0.038 mm silica spheres −one 4 mm glass bead	0.5 mm		0.7 mm	Mix of two types beads: 0.1 and 0.5 mm	1.4 mm
Chemical composition of beads		Glass beads		Garnet beads	ZR BashingBead TM: ultra-high density beads are fracture resistant, chemically inert	Ceramic beads
Duration of grinding	1 min	3 min		10 min	45 s	1 min
Speed of grinding	6 m/s	30 Hz (1800 oscillations/min)		3200 rpm	7000 rpm	3500 rpm
Homogenizer system (company)	High-speed benchtop homogenizer FastPrep 24 (MP Biomedicals)	High-speed benchtop homogenizer TissueLyser^®^ II (Qiagen)		Vortex homogenizer Vortex-Genie 2^®^ (Scientific industries)	High-speed benchtop homogenizer MagnaLyser^®^ (Roche Diagnostics)	
Lysis buffer (company) [volume]	Nuclisens^®^ easyMAG^®^ Lysis buffer (bioMérieux) [1 mL]	Nuclisens^®^ easyMAG Lysis buffer (bioMérieux) [800 µL]		PowerBead Solution QIAamp PowerFecal DNA kit (Qiagen) [750 µL]	BashingBead Buffer^®^, Quick DNA Fecal/Soil Microbe Microprep kit (ZymoResearch) [750 µL]	Bacterial Lysis Buffer (Roche Diagnostics) [500 µL]

EM, Nuclisens^®^ EasyMAG^®^, bioMérieux; IN, ELITE InGenius^®^; ELITechGroup; QF, QIAamp Fast DNA Stool Mini Kit^®^, Qiagen; QP, QIAamp Power Fecal DNA Kit^®^, Qiagen; ZR, Quick DNA Fecal/Soil Microbe Miniprep^®^, ZymoResearch; MPG, MagnaPure 96^®^ with grinding, Roche Diagnostics.

**Table 3:** Characteristics of the DNA extraction kits.

Participating Centers	Kits (Company)	Abbreviated Name	Extraction System	Stool Test Sample (µL)	Elution Volume (µL)	Type of Lysis	Mechanical Pretreatment	Purification Support/Technology	Maximum No. of Samples/Run
Center 3	1. QIAamp Power fecal DNA kit^®^ (Qiagen)	QP	M	250	100	C + T	Included Bead Tubes, Dry Garnet^®^ (Qiagen)	Silica column	
Center 2	2. QIAamp Fast DNA Stool Mini Kit^®^ (Qiagen)	QF	M	200	200	C + T + E	Not included	Silica column	
Center 4	3. Quick-DNA Fecal/Soil Microbe Miniprep^®^ (Zymo Research)	ZR	M	150	100	C	Included ZR BashingBead Lysis Tubes ^®^	Silica column	
Center 2	4. Elite Ingenius^®^ (ELITechGroup)	IN	A	200	A choice: 50 or 100	C	Not included	Magnetic silica/Magtration^®^ technology	12
Center 1	5. Nuclisens^®^ easyMAG^®^ with Nuclisens^®^ easyMAG^®^ Silice magnet (bioMérieux)	EM	A	400	100	C	Not included	Magnetic silica/Boom^®^ technology	24
Center 5	6. MagnaPure 96 System^®^ with MagNA Pure 96 DNA and Viral NA Small Volume^®^ (Roche Diagnostics)	MP: MagnaPure 96 without grinding MPG: MagnaPure 96 with grinding	A	200	100	C + T + E	Not included	Magnetic silica/technology based on the use of magnetic glass beads	96

M, manual; A, automated; T, thermic; E, enzymatic; C, chemical.

**Table 4:** Performances of six *C. parvum* DNA pretreatment/extraction protocols studied.

Pretreatment/Extraction Protocol	Proportion of Positive Samples at Each Concentration (%)					Overall Proportion of Positive Samples (%)
	10 oocysts/mL	50 oocysts/mL	100 oocysts/mL	500 oocysts/mL	1000 oocysts/mL	
QF	0	33.3	50	100	100	56.7
MP	44.4	66.7	88.9	100	100	80
IN	44.4	72.2	88.9	100	100	81.1
MPG	66.7	94.4	100	100	100	92.2
EM	66.7	100	100	100	100	93.3
QP	83.3	88.9	100	100	100	94.4
ZR	94.4	100	100	100	100	98.9

QF, QIAamp Fast DNA Stool Mini Kit^®^, Qiagen; MP, MagnaPure 96^®^ without grinding, Roche Diagnostics; IN, ELITE InGenius^®^, ELITechGroup; MPG, MagnaPure 96^®^ with grinding, Roche Diagnostics; EM, Nuclisens^®^ EasyMAG^®^, bioMérieux; QP, QIAamp Power Fecal DNA Kit^®^, Qiagen; ZR, Quick DNA Fecal/Soil Microbe Miniprep^®^, ZymoResearch.

**Table 5:** Degrees of statistical significance of Chi2 tests comparing the percentage of positive *C. parvum* real-time PCR according to the pretreatment/extraction protocols for the concentrations of 10, 50, and 100 oocysts/mL.

	EM			IN			QF			QP			ZR			MPG			MP		
	10	50	100	10	50	100	10	50	100	10	50	100	10	50	100	10	50	100	10	50	100
EM	na	na	na	ns	*	ns	***	***	***	ns	ns	ns	ns	ns	ns	ns	ns	ns	ns	**	ns
IN	ns	***	ns	na	na	na			**	*	ns	ns	**	*	ns	ns	ns	ns	ns	ns	ns
QF	***	ns	***	**	*	**	na	na	na			***	***	***	***	***	***	***	**	ns	**
QP	ns	ns	ns	*	ns	ns	***	**	***	na	na	na	ns	ns	ns	ns	ns	ns	*	ns	ns
ZR	ns	ns	ns	**	*	ns	***	***	***	ns	ns	ns	na	na	na	ns	ns	ns	**	***	ns
MPG	ns	ns	ns	ns	ns	ns	***	***	***	ns	ns	ns	ns	ns	ns	na	na	na	ns	ns	ns
MP	ns	**	ns	ns	ns	ns	**	ns	**	*	ns	ns	**	**	ns	ns	ns	ns	na	na	na

* (*p* ≤ 0.05), ** (*p* ≤ 0.01), *** (*p* ≤ 0.001), ns, not significant, na, not applicable. EM, Nuclisens^®^ EasyMAG^®^, bioMérieux; IN, ELITE InGenius^®^, ELITechGroup; QF, QIAamp Fast DNA Stool Mini Kit^®^, Qiagen; QP, QIAamp Power Fecal DNA Kit^®^, Qiagen; ZR, Quick DNA Fecal/Soil Microbe Miniprep^®^, ZymoResearch; MPG, MagnaPure 96^®^ with grinding, Roche Diagnostics; MP, MagnaPure 96^®^ without grinding, Roche Diagnostics.

**Table 6:** Degrees of statistical significance of Mann–Whitney tests comparing mean Ct values of *C. parvum* real-time PCR according to DNA pretreatment/extraction protocols for the concentrations of 100, 500 and 1000 oocysts/mL.

	EM			IN			QF			QP			ZR			MPG			MP		
	100	500	1000	100	500	1000	100	500	1000	100	500	1000	100	500	1000	100	500	1000	100	500	1000
EM	na	na	na	***	***	***	***	***	***	ns	ns	***	***	***	***	*	***	ns	*	***	***
IN	***	***	***	na	na	na	**	***	***	*	***	***	*	***	***	***	***	***	***	ns	***
QF	***	***	***	**	***	***	na	na	na	***	***	***	***	***	***	***	***	***	***	***	***
QP	ns	ns	***	*	***	***	***	***	***	na	na	na	***	***	***	*	***	***	**	***	***
ZR	***	***	***	*	***	***	***	***	***	***	***	***	na	na	na	***	***	***	***	***	***
MPG	*	***	ns	***	***	***	***	***	***	*	***	***	***	***	***	na	na	na	ns	***	***
MP	*	***	***	***	ns	***	***	***	***	**	***	***	***	***	***	ns	***	***	na	na	na

* (*p* ≤ 0.05), ** (*p* ≤ 0.01), *** (*p* ≤ 0.001), ns, not significant, na, not applicable. EM, Nuclisens^®^ EasyMAG^®^, bioMérieux; IN, ELITE InGenius^®^, ELITechGroup; QF, QIAamp Fast DNA Stool Mini Kit^®^, Qiagen; QP, QIAamp Power Fecal DNA Kit^®^, Qiagen; ZR, Quick DNA Fecal/Soil Microbe Miniprep^®^, ZymoResearch; MPG, MagnaPure 96^®^ with grinding, Roche Diagnostics; MP, MagnaPure 96^®^ without grinding, Roche Diagnostics.

**Table 7:** Average of Ct values and standard deviation of real-time PCR results according to the pretreatment/extraction protocol and to oocyst concentration.

Pretreatment/Extraction Protocol	Average of Ct Values ± Standard Deviation		
	100 oocysts/mL	500 oocysts/mL	1000 oocysts/mL
QF	37.60 ^a^ ± 1.03	35.99 ± 0.84	35.27 ± 0.84
IN	35.70 ^b^ ± 0.94	33.13 ± 0.23	32.66 ± 0.28
MP	34.20 ^c^ ± 0.45	33.23 ± 0.44	33.46 ± 0.57
MPG	34.30 ± 0.81	30.86 ± 0.25	31.11 ± 0.48
EM	33.60 ± 0.48	31.53 ± 0.32	30.86 ± 0.31
QP	33.54 ± 0.39	31.48 ± 0.24	29.88 ± 0.20
ZR	31.91 ± 0.18	29.86 ± 0.16	28.89 ± 0.37

^a^, average calculated on 9 values of Ct; ^b^, average calculated on 16 values of Ct; ^c^, average calculated on 15 values of Ct; QF, QIAamp Fast DNA Stool Mini Kit^®^, Qiagen; MP, MagnaPure 96^®^ without grinding, Roche Diagnostics; IN, ELITE InGenius^®^, ELITechGroup; MPG, MagnaPure 96^®^ with grinding, Roche Diagnostics; EM, Nuclisens^®^ EasyMAG^®^, bioMérieux; QP, QIAamp Power Fecal DNA Kit^®^, Qiagen; ZR, Quick DNA Fecal/Soil Microbe Miniprep^®^, ZymoResearch.
